# Cancer in Persons Working in Dry Cleaning in the Nordic Countries

**DOI:** 10.1289/ehp.8425

**Published:** 2005-10-13

**Authors:** Elsebeth Lynge, Aage Andersen, Lars Rylander, Håkan Tinnerberg, Marja-Liisa Lindbohm, Eero Pukkala, Pål Romundstad, Per Jensen, Lene Bjørk Clausen, Kristina Johansen

**Affiliations:** 1 Institute of Public Health, University of Copenhagen, Copenhagen, Denmark; 2 Cancer Registry of Norway, Oslo, Norway; 3 Department of Occupational and Environmental Medicine, Lund University Hospital, Lund, Sweden; 4 Finnish Institute of Occupational Health, Helsinki, Finland; 5 Finnish Cancer Registry, Institute for Statistical and Epidemiological Cancer Research, Helsinki, Finland; 6 Department of Community Medicine and General Practice, Norwegian University of Science and Technology, Trondheim, Norway

**Keywords:** cancer incidence, case–control study, dry cleaning, occupational exposure, tetrachloroethylene

## Abstract

U.S. studies have reported an increased risk of esophageal and some other cancers in dry cleaners exposed to tetrachloroethylene. We investigated whether the U.S. findings could be reproduced in the Nordic countries using a series of case–control studies nested in cohorts of laundry and dry-cleaning workers identified from the 1970 censuses in Denmark, Norway, Sweden, and Finland. Dry-cleaning work in the Nordic countries during the period when tetrachloroethylene was the dominant solvent was not associated with an increased risk of esophageal cancer [rate ratio (RR) = 0.76; 95% confidence interval (CI), 0.34–1.69], but our study was hampered by some unclassifiable cases. The risks of cancer of the gastric cardia, liver, pancreas, and kidney and non-Hodgkin lymphoma were not significantly increased. Assistants in dry-cleaning shops had a borderline significant excess risk of cervical cancer not found in women directly involved in dry cleaning. We found an excess risk of bladder cancer (RR = 1.44; 95% CI, 1.07–1.93) not associated with length of employment. The finding of no excess risk of esophageal cancer in Nordic dry cleaners differs from U.S. findings. Chance, differences in level of exposure to tetrachloroethylene, and confounding may explain the findings. The overall evidence on bladder cancer in dry cleaners is equivocal.

Previous studies of dry cleaners, primarily from the United States, indicated that exposure to tetrachloroethylene may cause an increased risk of cancer of the esophagus and cervix uteri and of non-Hodgkin lymphoma (NHL) [International Agency for Research on Cancer (IARC) 1995]. We investigated the incidence of selected cancers in Nordic dry cleaners to determine whether the U.S. findings could be reproduced in another setting.

The study was undertaken as a series of case–control studies nested in the cohorts of laundry and dry-cleaning workers identified from the 1970 censuses in Denmark, Norway, Sweden, and Finland. The cancer incidence of these cohorts has been reported previously (Andersen et al. 1999), and the Danish cohort has been used for a nested case–control study of liver and kidney cancer (Lynge et al. 1995). Use of tetrachloroethylene reached its peak in the Nordic countries around 1970 (Danmarks Statistik 2000a, 2000b, 2000c; Statistiska Centralbyrån 1995a, 1995b, 2000a, 2000b, 2000c; Statistisk Sentralbyrå 2000a, 2000b, 2000c; Tilastokeskus 2000a, 2000b, 2000c) (Figure 1); almost all of it was used for dry cleaning (Mikkelsen et al. 1983), and tetrachloroethylene was the dominant solvent in dry cleaning at the time (Anonymous 1968, 1971). Based on findings in previous studies, we included esophageal and cervical cancer and NHL (IARC 1995). We also included liver cancer found in tetrachloroethylene-exposed mice (IARC 1995), renal cell cancer found in workers exposed to trichloroethylene (Henschler et al. 1995), and bladder and pancreas cancer found in recent updates of U.S. cohorts (Blair et al. 2003; Ruder et al. 2001). Gastric cardia cancer was included because adenocarcinomas are on the increase in esophagus and cardia in some Western countries (Botterweck et al. 2000).

The purpose of this study was to determine whether dry-cleaning work in the Nordic countries around 1970, when tetrachloroethylene was the dominant dry-cleaning solvent, was associated with an increased risk of the selected cancers. We used the nested case–control design to avoid confounding from socioeconomic group and related lifestyle risk factors.

## Materials and Methods

### Study base, cases, and controls.

The cohorts included all laundry and dry-cleaning workers from the 1970 censuses in Denmark, Finland, Norway, and Sweden. They had either the occupation code “laundry and dry-cleaning worker” or the industry code “laundry and dry cleaning” (International Labour Office 1981; Statistical Office of the United Nations 1958) (Table 1). The cohorts consisted of 46,768 persons. Each person was followed up for death, emigration, and incident cancer based on linkage with the nationwide population, death, and cancer registries using unique personal identifiers.

The present study included incident cancers of the esophagus, gastric cardia, pancreas, cervix uteri, bladder, and kidney, as well as primary liver cancer and NHL (Table 2), from the beginning of follow-up, 9 November 1970 in Denmark and 1 January 1971 in the other countries, until the end of follow-up between 1997 and 2001. Cancer cases were identified using combined topography and morphology codes from the International *Classification of Diseases for Oncology* (Percy 1990).

Controls were randomly selected from the cohort using frequency match by country, sex, 5-year age group, and 5-year calendar period at the time of diagnosis of the case. For esophageal cancer, we selected controls equal to six times the number of cases. For the other cancer sites, three times the number of cases.

The registry part of this study was approved by each of the national data protection agencies. The interview part of this study was approved by the ethics committees in Norway and Sweden; after national legislation, all participants gave active informed consent before participating in the interview.

### Exposure categories.

On the basis of various data sources and without knowledge of their case–control status, we categorized cases and controls as follows: *a*) exposed persons explicitly described as dry cleaners and other workers in dry-cleaning shops with < 10 workers (the latter group was included because of the shared work tasks and physical proximity in small shops); *b*) other workers in dry-cleaning shops; *c*) unexposed laundry workers and other persons not working in dry cleaning; and *d*) unclassifiable.

Exposed cases and controls were categorized by length of employment in the shop where they worked in 1970. For practical reasons, we included only the period 1964–1979. Data on smoking and alcohol drinking were collected in Norway and Sweden (Table 3).

The person’s specific occupational task as dry cleaner or laundry worker at the 1970 census was written in free text on the original census form. These forms were retrieved from the National Archives in Denmark and Norway. The forms had not been stored in Finland and Sweden.

A blinded personal telephone interview, eventually with a next-of-kin, was undertaken with cases and controls in Norway and Sweden. The questionnaire asked about occupational tasks in 1970, and if this was dry cleaning, then about length of employment in the shop, size of the work force, solvents used, and smoking and drinking habits. In Norway, interviews were obtained with 57% of cases (72% with next-of-kin) and with 64% of controls (42% next-of-kin). In Sweden, interviews were obtained with 63% of cases (77% next-of-kin) and with 60% of controls (39% next-of-kin). One-fourth of interviewed next-of-kin was 1970 spouses, and one-third of non-interviewed subjects had no next-of-kin.

Denmark and Finland have nationwide databases with individual records on all paid pension scheme contributions, and we used these pension scheme data for this study. In Denmark, these data started for employees in 1964; we used these data to assess length of employment and size of the work force where the employees worked in 1970. In Finland, these data started in 1962 for employees and in 1970 for self-employed persons; the data were used to assess length of employment where the persons worked in 1970. Pension scheme data were found for 91% (151 of 166) of Danish records for employees in dry cleaning, with missing data for 5 employees explained by sick leave and so on at the 1970 census. Pension scheme data were found for 75% of Finnish records.

In Denmark, we used a biography of dry-cleaning shop owners (Hammershøj 1971) and the yellow pages of local telephone books for self-employed persons to assess length of employment, with 37% from the book, 57% from telephone books, and no data for 6%. Family workers were assumed to have worked for the same length as their spouses. We used the book (Hammershøj 1971) and pension scheme data for the self-employed persons’ shops to assess the size of the work force.

For Finland, we used the pension scheme data in combination with other sources (Anonymous 1984; Kyyronen et al. 1989) to assess type and size of company (Table 3). For Finland and Sweden, we coded as unexposed those cases and controls we assumed from the census codes not to be dry cleaners (e.g., “presser” in “textile industry”).

We identified 1,616 cases and 2,398 controls (Table 2). Together they represented 3,883 persons. For Denmark and Norway, about 20% of the records were classified as coming from the exposed dry-cleaner group and 70–80% came from the unexposed group (Table 4). For Finland and Sweden, respectively, 41% and 35% of the records were unclassifiable as to whether the persons had dry-cleaning work in 1970.

**Figure 1:**
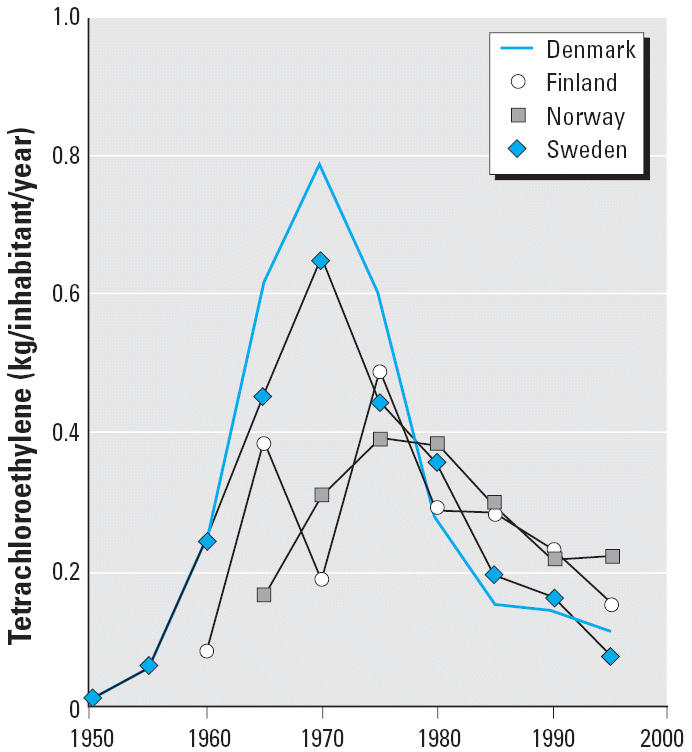
Use of tetrachloroethylene in the Nordic countries 1950–2000. The kilograms of tetrachloroethylene used in a given country was calculated as (kg manufactured + kg imported – kg exported). For calculation of kilograms per inhabitant per year, we divided the average tetrachloroethylene used in a 5-year period by the population size in the middle of the period.

Use of tetrachloroethylene peaked in the Nordic countries around 1970, and the compound was used almost exclusively for dry cleaning (Figure 1). In Denmark, import of the new fully automated German and English machines using tetrachloroethylene started in 1959 (Direktoratet for Arbejdstilsynet 1959). In 1967, 30% of conventional shops had machines obtained within the last 10 years (Schleisner 1967), and new coin-operated machines using only tetrachloroethylene made up 40% of the market in 1968 (Anonymous 1968).

In 1968, tetrachloroethylene constituted 75% of the solvents used for dry cleaning in Denmark, 85% in Finland, and 72% in Sweden (Anonymous 1968); in 1971 it was estimated to constitute 90% of dry-cleaning solvent used in Scandinavia (Anonymous 1971). In the questionnaires, 76% of dry cleaners in Norway and 84% in Sweden reported use of tetrachloroethylene in 1970, but information on chemicals and time periods was missing in many interviews. Tetrachloroethylene was thus clearly the dominant dry-cleaning solvent throughout our study period. Work as a dry cleaner in 1970 was therefore a good proxy for exposure to tetrachloroethylene, which is the underlying exposure variable of interest in this study. The probability of being exposed to tetrachloroethylene outside dry cleaning was extremely low because virtually all tetrachloroethylene was used in this industry (Mikkelsen et al. 1983). Available data did not allow further subdivision of dry cleaners as to whether or not they had used tetrachloroethylene. Other solvents in use were white spirit and chlorofluorocarbons (Johansen et al. 2005).

**Figure 2:**
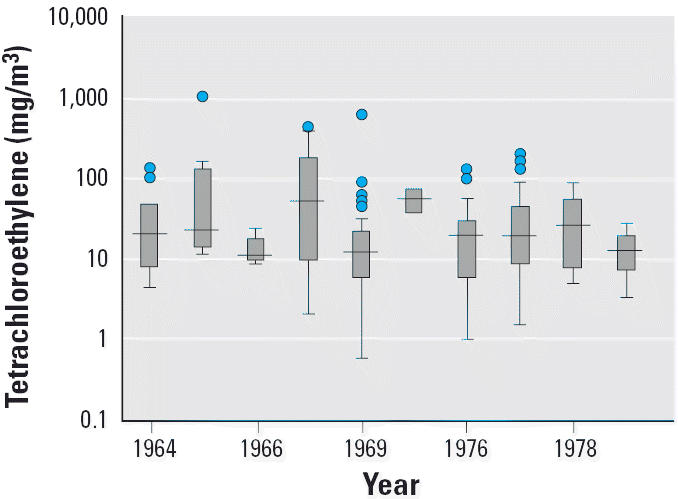
Tetrachloroethylene exposure in Nordic dry-cleaning shops 1964–1979. The solid line indicates median; bottoms and tops of boxes indicate 25th and 75th percentiles, respectively; bottom and top error bars indicate range, respectively; and circles indicate outliers.

In 1970, the occupational safety limit for tetrachloroethylene was 670 mg/m^3^ in Finland, 350 mg/m^3^ in Denmark and Norway, and 200 mg/m^3^ in Sweden. In 1980, these limits were 335, 200, and 135 mg/m^3^, respectively. Only 168 tetrachloroethylene measurements were made in dry-cleaning shops in the Nordic countries between 1964 and 1979. There was a large variation in exposure level across shops; the median annual level of all measurements was, however, fairly stable during 1964–1979 (Figure 2). In the analysis, we therefore assumed exposure level to tetrachloroethylene to be constant from 1964 to 1979 and used length of employment as a proxy for relative, cumulated dose. For comparison with external data, the mean of 53 measurements of ≥ 60 min for dry cleaners was 164 mg/m^3^.

### Analysis.

The analysis was based on records for cases and controls, because a given person could appear more than once. For a given cancer site, we used all controls fulfilling the selection criteria in the analysis. We estimated rate ratios (RRs) for dry cleaners versus unexposed controls using logistic regression adjusted for matching criteria and, where relevant, for smoking and alcohol use. For a comprehensive reporting of the data, we also calculated the RRs for the other persons in dry cleaning and for the unclassifiable persons, although the underlying hypothesis did not include these groups. RRs were estimated for all countries together and for Denmark and Norway together. We calculated RRs for the exposed group by length of employment. We used the R survival package (R Development Core Team 2004; Therneau and Lumbley 2004) for these analyses.

## Results

Eight esophageal cancer cases belonged to the dry-cleaner group, giving an RR of 0.76 [95% confidence interval (CI), 0.34–1.69] (Table 5). The estimate for Denmark and Norway gave an RR of 0.91 (95% CI, 0.38–2.20). Six exposed cases came from Denmark. Eighteen cases were unclassifiable, giving an RR of 2.04 (95% CI, 0.91–4.62); nine cases came from Finland (seven with missing pension scheme record) and nine non-interviewed cases came from Sweden. Nine gastric cardia cancer cases belonged to the dry-cleaner group, giving an RR of 0.69 (95% CI, 0.31–1.53).

Eleven exposed liver cancer cases gave an RR of 0.76 (95% CI, 0.38–1.52), and 57 exposed pancreatic cancer cases gave an RR of 1.27 (95% CI, 0.90–1.80). The highest risks were found for those with short or unknown length of employment (Table 6). Thirty-six exposed cervical cancer cases gave an RR of 0.98 (95% CI, 0.65–1.47), with the highest risk for those with short length of employment. There was a borderline significantly elevated risk of cervical cancer among other workers in dry-cleaning shops based on 22 cases, with an RR of 1.73 (95% CI, 1.00–2.97). Eleven cases were Danish (four pressers, three shop assistants, three office workers, one seamstress), seven were Finnish (six in laundries where dry cleaning was probable, one packer in a dry-cleaning shop of unspecified size), and four were Norwegian (two shop assistants, one laundry help, one spot cleaner).

Twenty-nine kidney cancer cases belonged to the dry-cleaner group, giving an RR of 0.67 (95% CI, 0.43–1.05). There was an elevated risk of bladder cancer among the dry cleaners based on 93 exposed cases (RR = 1.44; 95% CI, 1.07–1.93), with 62 exposed cases coming from Denmark and Norway, giving an RR of 1.69 (95% CI, 1.18–2.43). The risk did not increase with length of employment. Significantly elevated risks were found for 2–4 years and ≥ 10 years of employment. A similar pattern was seen when the analysis was based only on the uncensored employment periods from 1965 through 1978. The combined estimate for interviewed cases and controls from Norway and Sweden was RR = 1.34 (95% CI, 0.86–2.08), which was only slightly reduced after control for smoking (RR = 1.25; 95% CI, 0.79–1.98). The excess risk within the exposed group did not come from the owners of dry-cleaning shops and their employed dry cleaners (33 exposed cases, RR = 0.98; 95% CI, 0.64–1.51) but from the supporting staff in small shops (17 exposed cases, RR = 2.20; 95% CI, 1.18–4.11) and from owners of combined laundry and dry-cleaning shops (40 exposed cases, RR = 1.92; 95% CI, 1.23–2.98). There were 42 exposed NHL cases, giving an RR of 0.95 (95% CI, 0.65–1.41).

## Discussion

We studied the cancer risk in Nordic dry cleaners during the period where tetrachloroethylene was by far the dominant solvent, and we used laundry workers as the comparison group. Dry-cleaning work was not associated with an increased risk of esophageal cancer, but we found a borderline increased risk among persons we were unable to classify as dry cleaners or laundry workers. Dry-cleaning work was not associated with significantly increased risks of cancer of the gastric cardia, liver, pancreas, or kidney or with NHL. Female supportive staff in large dry-cleaning shops had a borderline significant excess risk of cervical cancer not found among women directly involved in dry cleaning. We found a 44% excess risk of bladder cancer among Nordic dry cleaners. The excess risk came from Denmark and Norway, the two countries with the best data. There was no clear pattern with length of employment. Adjustment for smoking in Norway and Sweden changed the estimated risk only slightly. The risk was concentrated among supporting staff in small dry-cleaning shops and among owners of combined laundry and dry-cleaning shops.

### Strengths and weaknesses of the study.

Our study had several advantages. First, we covered a period where tetrachloroethylene was the dominant solvent. Second, the study was nationwide, including all persons working in dry cleaning in 1970. Third, we used a series of case–control studies nested in the national cohorts of laundry and dry-cleaning workers. The cancer risks of dry cleaners were therefore compared with those of laundry workers, two groups with similar jobs apart from the use of solvents. Smoking was equally frequent among exposed (72%) and unexposed (78%) male controls in Norway, and equally so in Sweden (66% and 69%). In Norway, smoking was slightly less frequent in exposed (45%) than in unexposed (54%) women, whereas the opposite was true in Sweden (49% and 37%). Alcohol drinking was very limited, with only 4 of 675 interviewed controls reporting at least 21 drinks/week. Fourth, population, death, and cancer registries and unique personal identifiers ensured complete ascertainment of incident cancers (Pukkala et al. 2001). Fifth, all original census forms were found in Denmark and Norway, and they all included detailed job descriptions.

The study did, however, also have disadvantages. First, because of the limited data sources and mixture of processes, a high proportion of cases and controls from Sweden and Finland were unclassifiable as to whether they had dry-cleaning or laundry work in 1970. We therefore reported risk estimates for all countries and for Denmark and Norway only. Second, data on employment were available only from 1964 through 1979, but the 16-year period allowed a clear distinction to be made between short-term and stable workers. Third, the limited number of air measurements did not allow subdivision of study subjects by exposure level. However, because the data indicated a fairly stable exposure level throughout the study period, duration of employment was an acceptable proxy measure for relative cumulated dose.

### Esophageal cancer.

There was a clear excess risk of esophageal cancer in the two U.S. cohort studies of tetrachloroethylene-exposed dry-cleaning workers, with standardized mortality ratios (SMRs) of 2.2 (95% CI, 1.5–3.3; Blair et al. 2003) and 2.47 (95% CI, 1.35–3.14; Ruder et al. 2001), respectively. A non-significantly elevated risk was seen in the U.S. aircraft manufacturing workers exposed to tetrachloroethylene (SMR = 1.47; 95% CI, 0.54–3.21; Boice et al. 1999). Two dry cleaners with squamous cell carcinoma of the esophagus were found in a U.S. case–control study [odds ratio (OR) = 3.6; 95% CI, 0.5–27.0] (Vaughan et al. 1997).

Our estimated risk of esophageal cancer after dry-cleaning work in the Nordic countries of RR = 0.76 (95% CI, 0.34–1.69) is in contrast with the U.S. findings (Blair et al. 2003, Ruder et al. 2001), although the difference in the outcome of the four studies could be due to chance. No case of esophageal cancer was found in a small Finnish cohort (Anttila et al. 1995). Unfortunately, in our study 18 cases were unclassifiable, and they had a statistically nonsignificantly increased risk (RR = 2.04; 95% CI, 0.91–4.62). We know little about these cases. However, even in the extreme and unlikely situation where all unclassifiable persons were exposed, our risk estimate would be RR = 1.19 (95% CI, 0.67–2.12). If all unclassifiable persons were unexposed, our risk estimate for the exposed group would be RR = 0.66 (95% CI, 0.30–1.45).

The excess risk of esophageal cancer in U.S. dry cleaners (Blair et al. 2003, Ruder et al. 2001) but not found in Nordic dry cleaners may be due to chance, different confounders, and/or different exposures. Esophageal cancer is associated with smoking, alcohol consumption, hot drinks, and poor nutrition (Muños and Day 1996). The mortality of the U.S. dry cleaners (Blair et al. 2003, Ruder et al. 2001) was compared with that of the national population, without control for possible confounders. However, national smoking data showed laundry and dry-cleaning workers to be only marginally more frequent smokers than the general U.S. population (Blair et al. 2003; Ruder et al. 2001), but the average earning of dry cleaners was only two-thirds of the average for private sector workers (Blair et al. 2003). We used laundry workers with similar jobs apart from the solvents as the comparison group. The self-employed Danish dry cleaners were members of Lions Club, Rotary, and so forth (Hammershøj 1971).

In 1991, about one-third of U.S. dry-cleaning plants used an open transfer process where solvent-wet clothes were manually moved from washer to dryer (Mundt et al. 2003). Based on large U.S. samples of time-weighted-average measurements for machine operators from the 1980s, the exposure level was higher at transfer machines than at dry-to-dry machines: mean concentrations were 338 mg/m^3^ and 157 mg/m,^3^ respectively (IARC 1995). This transfer process was not needed in the Danish, widely exported, semiautomated machines used since the 1930s (Ingvordsen 1975), and manual handling of wet clothes became prohibited in 1953 (Arbejds-og Fabrikstilsynet 1953). The mean concentration of Nordic measurements ≥ 60 min for machine operators from 1980 through 1990 was 95 mg/m^3^. The currently recommended threshold from the American Conference of Governmental Industrial Hygienists is 170 mg/m^3^ [Occupational Safety and Health Administration (OSHA) 2005], whereas the current safety limit is 70 mg/m^3^ in Denmark, Finland, and Sweden and 40 mg/m^3^ in Norway (Arbejdstilsynet 2002, 2003; Ministry of Social Affairs and Health 2005; Swedish National Board of Occupational Safety and Health 1997). U.S. dry cleaners thus had a higher probability of dermal tetrachloroethylene exposure than did Nordic dry cleaners, and they were very probably exposed to a higher air concentration. Differences in exposure to tetrachloroethylene along with differences in socioeconomic status may therefore have contributed to the excess risk of esophageal cancer found in U.S. but not in Nordic dry cleaners.

### Other cancers.

Data on primary liver cancer were reported in only two U.S. studies (Blair et al. 2003; Ruder et al. 2001) with no excess risk. This is in line with the present result.

One U.S. dry-cleaner cohort had a borderline excess risk of pancreatic cancer (SMR = 1.53; 95% CI, 0.91–2.42; Ruder et al. 2001), as did aircraft manufacturing workers (SMR = 1.50; 95% CI, 0.72–2.76; Boice et al. 1999). However, the other U.S. dry-cleaner cohort (Blair et al. 2003), the Finnish cohort (Anttila et al. 1995), and the present study did not confirm this finding.

The two U.S. dry-cleaner cohorts had excess risks of cervical cancer (Ruder et al. 2001: SMR = 1.95; 95% CI, 1.00–3.40; Blair et al. 2003: SMR = 1.6; 95% CI, 1.0–2.3), an observation confirmed in the Finnish cohort based on small numbers (Anttila et al. 1995) but not among the U.S. aircraft workers (Boice et al. 1999). In U.S. dry cleaners, the risk was increased both for work with tetrachloroethylene only and for mixed solvents (Ruder et al. 2001), and the risk did not vary with exposure status (Blair et al. 2003). In our study, dry cleaners had no excess risk of cervical cancer (RR = 0.98; 95% CI, 0.65–1.47). There was, however, a borderline significant elevated risk among supporting staff in larger dry-cleaning shops (RR = 1.73; 95% CI, 1.00–2.97). We thus confirmed previous findings of an excess risk of cervical cancer among women in dry-cleaning shops, but the fact that they were not engaged in the dry-cleaning process did not point to tetrachloroethylene as the explanatory risk factor, nor did it point to social class, because the comparison group was laundry workers.

Kidney cancer was not increased in the previous cohort studies (Blair et al. 2003; Boice et al. 1999; Ruder et al. 2001) or in our study.

The risk of bladder cancer was increased in one U.S. dry-cleaner cohort (SMR = 2.22; 95% CI, 1.06–4.08; Ruder et al. 2001) but not in the other (SMR = 1.3; 95% CI, 0.7–2.4; Blair et al. 2003) and not in aircraft workers (Boice et al. 1999). The Finnish study did not report on bladder cancer (Anttila et al. 1995). The excess risk in the United States was limited to those working with mixed solvents (Ruder et al. 2001), found only in whites, and equally so in those with little or no exposure and those with medium or exposure (Blair et al. 2003). The U.S. bladder cancer case–control study reported an excess risk for dry-cleaning work in non-white men (OR = 2.80; 95% CI, 1.10–7.40; Silverman et al. 1989a) but not in white women (OR = 1.40; 95% CI, 0.80–2.50; Silverman et al. 1990), and data were not reported for white men (Silverman et al. 1989b). The risks for all laundry and dry cleaners of both sexes and races were 1.31 (95% CI, 0.85–2.03) for nonsmokers, 2.99 (95% CI, 1.80–4.97) for former smokers, and 3.94 (95% CI, 2.39–6.51) for current smokers (Smith et al. 1985). The joint analysis of European case–control studies showed a smoking-adjusted RR of 1.24 (95% CI, 0.67–2.31) for male launderers, dry cleaners, and pressers (Kogevinas et al. 2003). The case–control study from Montreal, Canada, gave an RR of 1.6 (90% CI, 0.9–3.1) for launderers and dry cleaners, but the risk was not elevated for exposure to tetrachloroethylene (Siemiatycki 1991). We found an elevated bladder cancer risk among dry cleaners (RR = 1.44; 95% CI, 1.07–1.93) that did not increase with length of employment. Taking the studies together, there appears to be an excess risk of about 45%, which does not seem to be explained by excessive smoking. The risk does not vary with the exposure indices. Overall, the current picture of the association between dry-cleaning work with tetrachloroethylene and risk of bladder cancer is equivocal.

In a 1995 monograph on dry cleaning (IARC 1995), an excess risk of NHL was described based on studies then available (Anttila et al. 1995; Blair et al. 1990; Boice et al. 1999). However, whereas the previous analysis of the largest cohort included only *International Classification for Diseases*, version 8 [ICD-8; World Health Organization (WHO) 1965] code 200 (Blair et al. 1990), the update included ICD-8 codes 200 and 202 (Blair et al. 2003), showing no excess risk. At present, the three studies together give 22 observed cases and 18.80 expected. Our results are in line with this.

## Conclusion

Dry-cleaning work in the Nordic countries, during a period when tetrachloroethylene was the dominant solvent, was not associated with significantly increased risks of cancer of the gastric cardia, pancreas, or kidney or with primary liver cancer or NHL. Dry-cleaning work was not associated with an increased risk of esophageal cancer, but our study was hampered by some unclassifiable cases. The result for esophageal cancer contrasts findings from U.S. tetrachloroethylene-exposed cohorts, which could be due to chance, confounding, or differences in exposure level. In line with findings from previous studies, our study indicated an excess risk of cervical cancer in supporting staff in larger dry-cleaning shops, but not in women directly involved in dry cleaning. We found an elevated risk of bladder cancer among Nordic dry cleaners. The international data together point to an excess risk of bladder cancer in dry cleaners of about 45%, but there is no pattern with exposure indices. The evidence for an association between exposure to tetrachloroethylene and risk of bladder cancer is equivocal.

## Figures and Tables

**Table 1:** Industry and occupation codes in the 1970 censuses used for selection of the cohort of laundry and dry-cleaning workers in the Nordic dry-cleaner study.

	Occupation		Industry		
Country	Code	Description	Code	Description	No. of persons
Denmark	411^a^	Laundry worker, ironer	860^b^	Laundry, dry-cleaning	15,559
Finland	85^c^	Laundry and pressing	952^b^	Laundry service	6,885
Norway	95^c^	Laundering, dry-cleaning and pressing work	931^b^	Laundries and laundry service, cleaning and drying	6,874
Sweden	943^c^ 944^c^	Laundry and dry-cleaning work, pressing work	9,520^b^	Laundry and dry-cleaning service	17,450
Total					46,768

a Special Danish occupational code (Danmarks Statistik 1974).

b International Standard Industrial Classification (Statistical Office of the United Nations 1958).

c Nordic Occupational Classification, which is equivalent to the International Standard Classification of Occupations (International Labour Office 1981).

**Table 2:** Cancer cases and selected controls identified in the Nordic dry-cleaner study.

			Men					Women					
Cancer site	Topography	Morphology	Denmark	Finland	Norway	Sweden	Total	Denmark	Finland	Norway	Sweden	Total	All^a^
Esophagus	C15.0–C15.9	8000–8580^b^	15	2	3	6	26	19	12	5	10	46	72
Gastric cardia	C16.0	8000–8580^b^	10	1	2	16	29	7	4	4	6	21	50
Liver, primary	C22.0–C22.1	8000–8580^b^	9	2	2	10	23	26	16^c^	4	26	72	95
Pancreas	C25	8000–8580^b^	26	5	14	19	64	74	39	39	83	235	299
Cervix uteri	C53.0–C53.9	8000–8580^b^						128	29	44	87	288	288
Kidney	C64.9	8312.3	17	3	12	24	56	37	21	19	77	154	210
Bladder	C67	8000–8580^b^	71	4	32	70	177	60	20^c^	36	60	176	353
NHL	All	9590–9595, 9670–9698, 9711–9723^b^	18	7^c^	12	30	67	42	48^c^	30	62	182	249
Total cases			166	24	77	175	442	393	189	181	411	1,174	1,616
Controls			294	72^d^	160	291	817	537	282^d^	297	465	1,581	2,398

a In total, 3,883 subjects, because a given subject can be included more than once.

b Behavior code 3 only.

c One male NHL, one female liver, two female bladder, and one female NHL have been excluded from the analysis because there was no matching control.

d Twelve male controls and six female controls have been excluded from the analysis because there was no matching case. Topography and morphology codes based on Percy (1990).

**Table 3:** Data sources used for the exposure classification in the Nordic dry-cleaner study.

Variable	Denmark	Finland	Norway	Sweden
Inclusion in the study	1970 census	1970 census	1970 census	1970 census
Occupation code in 1970	Computerized census data	Computerized census data	Computerized census data	Computerized census data
Industry code in 1970	Computerized census data	Computerized census data	Computerized census data	Computerized census data
Detailed occupation in 1970	Census forms	No data	Census forms	Interviews
Detailed industry in 1970	Census forms plus other sources^a^	Pension schemes	Census forms	Interviews
Size of the workplace where the person worked in 1970	Employees: pension schemes Self-employed plus family workers: industry book plus pension schemes	Pension schemes plus other sources^a^	Interviews	Interviews
Length of employment in the workplace where the person worked in 1970	Employees: pension schemes Self-employed plus family workers: industry book plus telephone books^b^	Pension schemes	Interviews	Interviews
Tobacco smoking and alcohol intake	No data	No data	Interviews	Interviews

a Questionnaire data on shop characteristics collected from employers in 1984 for a study on tetrachloroethylene and reproductive outcome (Kyyronen et al. 1989), records of persons biologically monitored for exposure at the Finnish Institute of Occupational Health, register of industrial hygiene measurements from the same institute, yearly calendars of the Finnish Association of Laundry and Dry Cleaning Employers, and a directory of Finnish companies and company facilities (Anonymous 1984).

b All shops had a telephone, and the telephone book, in most cases, listed the telephone number together with both the name of the shop and the name of the shop owner.

**Table 4:** Cases and controls in the Nordic dry-cleaner study by country and exposure category.

	Denmark	Finland	Norway	Sweden	Total
Exposure category	No. (%)	No. (%)	No. (%)	No. (%)	No. (%)
Unexposed	1,088 (78)^a^	234 (41)	498 (70)^b^	600 (45)	2,420 (60)
Dry cleaner and other exposed	244 (18)	41 (7)	153 (21)	257 (19)	695 (17)
Other in dry cleaning	58 (4)	62 (11)	51 (7)	12 (1)	183 (5)
Unclassifiable	0 (0)	230 (41)	13 (2)	473 (35)	716 (18)
Total	1,390 (100)	567 (100)	715 (100)	1,342 (100)	4,014 (100)

a Includes 12 original forms erroneously coded as laundry and dry-cleaning workers in the 1970 census.

b Includes 55 original forms erroneously coded as laundry and dry-cleaning workers in the 1970 census.

**Table 5:** RRs for studied cancer sites for dry cleaners in the Nordic countries 1970–2000 in the Nordic dry-cleaner study.

	Denmark, Finland, Norway, and Sweden				Denmark and Norway only			
Cancer site	Unexposed	Dry-cleaner^a^	Other in dry-cleaning	Unclassifiable	Unexposed	Dry-cleaner^a^	Other in dry-cleaning	Unclassifiable
Esophagus								
Cases (*n*)	41	8	5	18	33	7	2	0
Controls (*n*)	342	86	31	108	242	55	20	1
RR	1	0.76	1.22	2.04	1	0.91	0.66	NR
95% CI	NR	0.34–1.69	0.41–3.63	0.91–4.62	NR	0.38–2.20	0.14–3.01	NR
Gastric cardiac								
Cases (*n*)	31	9	1	9	19	4	0	0
Controls (*n*)	201	80	8	68	125	42	7	0
RR	1	0.69	0.84	0.76	1	0.51	NR	NR
95% CI	NR	0.31–1.53	0.10–7.10	0.31–1.90	NR	0.16–1.62	NR	NR
Liver								
Cases (*n*)	58	11	2	23	36	4	1	0
Controls (*n*)	398	95	22	121	248	42	15	1
RR	1	0.76	0.42	1.11	1	0.62	0.41	NR
95% CI	NR	0.38–1.52	0.09–1.89	0.59–2.09	NR	0.21–1.89	0.05–3.25	NR
Pancreas								
Cases (*n*)	173	57	18	51	109	32	10	2
Controls (*n*)	769	206	59	242	512	112	42	1
RR	1	1.27	1.26	0.87	1	1.38	1.06	6.17
95% CI	NR	0.90–1.80	0.70–2.26	0.59–1.31	NR	0.87–2.20	0.50–2.25	0.56–68.21
Cervix								
Cases (*n*)	186	36	22	44	136	19	15	2
Controls (*n*)	744	150	51	186	516	77	34	3
RR	1	0.98	1.73	1.11	1	0.92	1.64	2.62
95% CI	NR	0.65–1.47	1.00–2.97	0.72–1.71	NR	0.54–1.59	0.87–3.11	0.42–16.26
Kidney								
Cases (*n*)	129	29	9	43	63	15	6	1
Controls (*n*)	589	196	34	241	342	99	21	3
RR	1	0.67	1.15	0.76	1	0.77	1.50	1.22
95% CI	NR	0.43–1.05	0.52–2.53	0.50–1.16	NR	0.41–1.44	0.55–4.08	0.12–12.11
Bladder								
Cases (*n*)	189	93	12	57	129	62	7	0
Controls (*n*)	904	292	52	234	639	173	38	3
RR	1	1.44	1.08	1.24	1	1.69	1.13	NR
95% CI	NR	1.07–1.93	0.55–2.11	0.83–1.83	NR	1.18–2.43	0.51–2.50	NR
NHL								
Cases (*n*)	145	42	8	52	83	16	3	0
Controls (*n*)	720	219	48	255	424	107	25	2
RR	1	0.95	0.70	0.91	1	0.73	0.64	NR
95% CI	NR	0.65–1.41	0.31–1.55	0.61–1.36	NR	0.40–1.32	0.19–2.23	NR

NR, not relevant.

a Includes persons stated to be dry cleaners, owners of dry-cleaning shops, and other persons employed in dry-cleaning shops with < 10 workers.

**Table 6:** RRs for the studies cancer sites in dry cleaners in the Nordic countries 1970–2000 by length of employment in the Nordic dry-cleaner study.

		Dry cleaner:^a^ length of employment				
Cancer site	Unexposed	0–1 year	2–4 years	5–9 years	≥ 10 years	Unknown
Esophagus						
Cases (*n*)	41	0	1	3	3	1
Controls (*n*)	261	0	5	29	27	4
RR	1	NR	1.20	0.66	0.70	1.65
95% CI	NR	NR	0.14–10.41	0.19–2.29	0.20–2.49	0.18–14.98
Gastric cardiac						
Cases (*n*)	31	0	0	2	6	1
Controls (*n*)	189	4	5	26	36	2
RR	1	NR	NR	0.46	0.97	3.00
95% CI	NR	NR	NR	0.10–2.02	0.36–2.58	0.24–38.19
Liver						
Cases (*n*)	58	0	0	5	5	1
Controls (*n*)	359	5	7	26	45	2
RR	1	NR	NR	1.21	0.70	2.88
95% CI	NR	NR	NR	0.43–3.44	0.26–1.92	0.21–38.81
Pancreas						
Cases (*n*)	172	6	7	14	23	7
Controls (*n*)	707	12	19	52	88	13
RR	1	2.14	1.38	1.18	1.20	2.44
95% CI	NR	0.76–6.06	0.54–3.50	0.62–2.25	0.72–1.99	0.90–6.66
Cervix						
Cases (*n*)	185	7	6	6	16	1
Controls (*n*)	678	8	26	47	50	3
RR	1	2.68	0.78	0.47	1.18	1.14
95% CI	NR	0.89–8.11	0.31–1.94	0.20–1.13	0.64–2.15	0.12–11.00
Kidney						
Cases (*n*)	125	1	4	8	14	2
Controls (*n*)	505	12	19	47	71	11
RR	1	0.24	0.86	0.70	0.75	0.70
95% CI	NR	0.03–2.04	0.28–2.67	0.32–1.55	0.39–1.42	0.15–3.36
Bladder^b^						
Cases (*n*)	188	6	10	17	53	6
Controls (*n*)	826	17	21	80	135	14
RR	1	1.50	2.39	0.91	1.57	1.97
95% CI	NR	0.57–3.96	1.09–5.22	0.52–1.59	1.07–2.29	0.64–6.05
NHL						
Cases (*n*)	145	5	3	14	15	5
Controls (*n*)	632	13	18	60	94	14
RR	1	1.35	0.61	0.92	0.66	1.47
95% CI	NR	0.44–4.14	0.17–2.21	0.49–1.72	0.36–1.22	0.49–4.47

NR, not relevant.

a Includes persons stated to be dry cleaners, owners of dry-cleaning shops, and other persons employed in dry-cleaning shops with < 10 workers.

b Analysis based only on the uncensored employment periods from 1965 through 1978 gave the following RRs: 0–1 year = 1.43 (95% CI, 0.52–3.97); 2–4 years = 2.38 (95% CI, 1.08–5.24); 5–9 years = 1.21 (95% CI, 0.58–2.50); ≥ 10 years = 2.84 (95% CI, 0.97–8.35); unknown = 2.12 (95% CI, 0.65–6.85).
